# Supplementary material for: Fully bioresorbable hybrid opto-electronic neural implant system for simultaneous electrophysiological recording and optogenetic stimulation
Source: Nat Commun. 2024 Mar 6;15:2000. doi: 10.1038/s41467-024-45803-0 (PMC10917781; doi:10.1038/s41467-024-45803-0)
Supplement: Supplementary file 1 — Supplementary information [file 41467_2024_45803_MOESM1_ESM.pdf]

Fully Bioresorbable Hybrid Opto-Electronic Neural Implant System for Simultaneous Electrophysiological Recording and Optogenetic Stimulation

Myeongki Cho†, Jeong-Kyu Han†, Jungmin Suh †, Jeong Jin Kim, Jae Ryun Ryu, In Sik Min, Mingyu Sang, Selin Lim, Tae Soo Kim, Kyubeen Kim, Kyowon Kang, Kyuhyun Hwang, Kanghwan Kim, Eun-Bin Hong, Min-Ho Nam, Jongbaeg Kim, Young Min Song, Gil Ju Lee*, Il-Joo Cho*, Ki Jun Yu*

*Corresponding authors: gjlee0414@pusan.ac.kr (G.J.L.), ijcho@korea.ac.kr (I-J.C.), and kijunyu@yonsei.ac.kr (K.J.Y.)
†These authors contributed equally to this work.

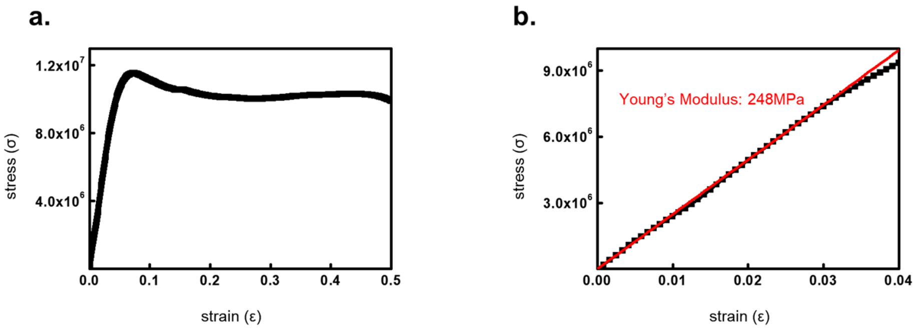

Supplementary Figure S1 | Stress-strain curves of the PLGA film. (a) Elastic-plastic property of PLGA film. (b) Calculated Young's modulus of PLGA film by linear fitting.

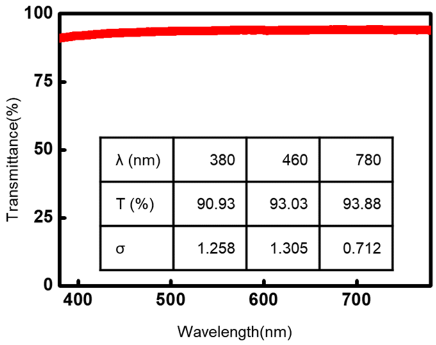

Supplementary Figure S2 | Visible light transmittance spectra of the PLGA film.

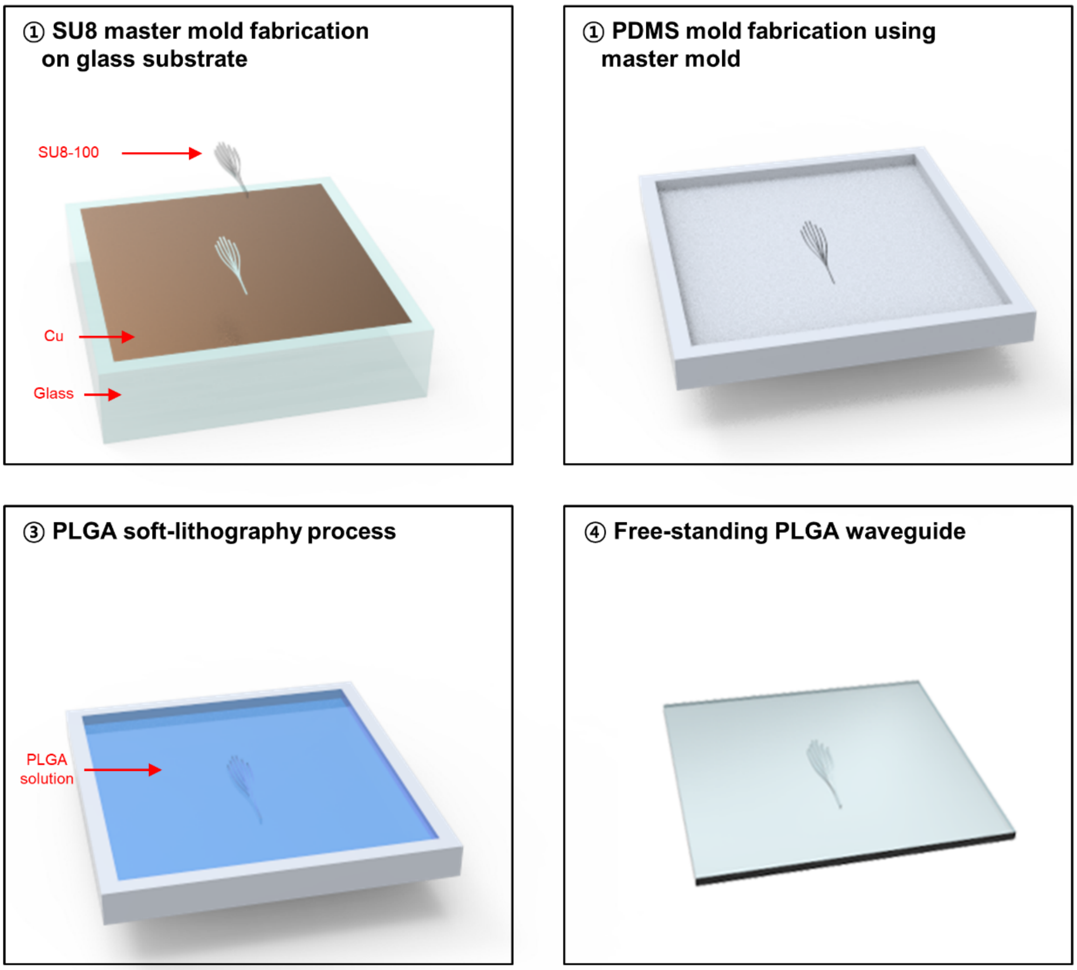

Supplementary Figure S3 | Materials and fabrication process of the bioresorbable PLGA waveguide.

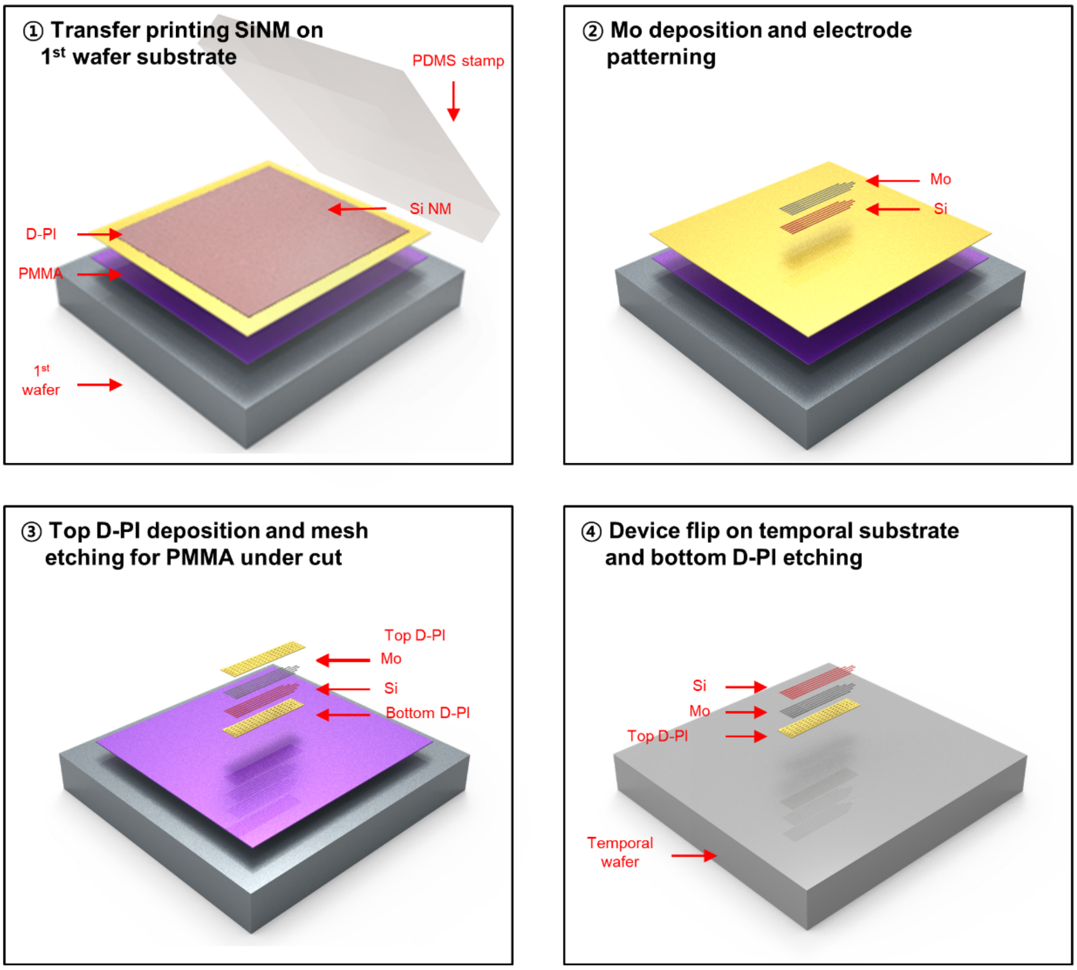

Supplementary Figure S4 | Materials and fabrication process of the bioresorbable Si/Mo electrode.

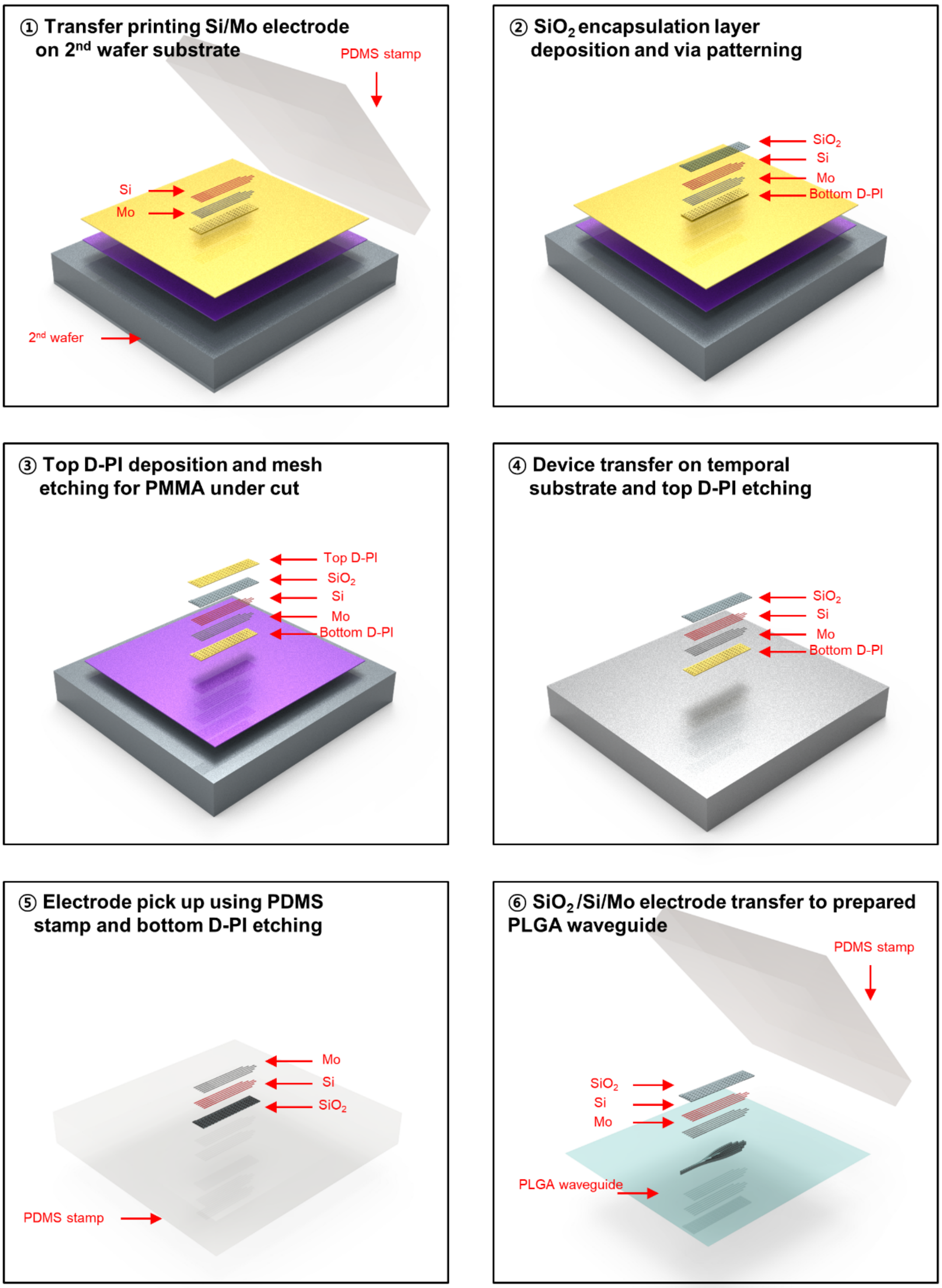

Supplementary Figure S5 | Materials and fabrication process of the bioresorbable SiO_2/Si/Mo electrode and device integration.

Wavelength	404 nm	532 nm	632.8 nm	829 nm
$n_{PLGA\ 75:25}$	1.4750	1.4653	1.4600	1.4539
$\sigma_{PLGA\ 75:25}$	0.00183	0.00175	0.00093	0.00057
$n_{PLGA\ 50:50}$	1.4787	1.4692	1.4636	1.4569
$\sigma_{PLGA\ 50:50}$	0.00025	0.00121	0.00105	0.00045

(Thickness of film : 100 μm, 23℃, n=3)

Supplementary Figure S6 | Mean and standard deviation values of PLGA film refractive index as a function of composition.

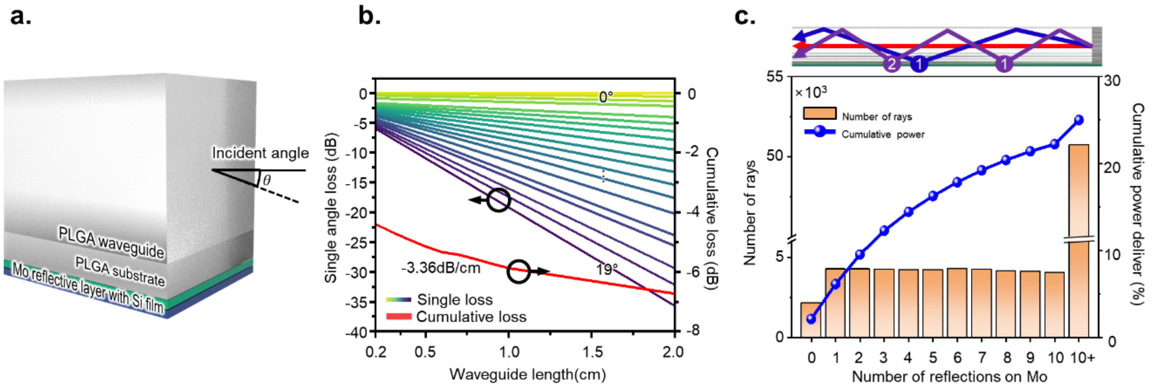

Supplementary Figure S7 | The single-channel waveguide simulation with Mo reflective layer. (a) Simulation system to measure loss of single-channel waveguide. The waveguide consists of PLGA waveguide, PLGA substrate, Mo reflective layer and Si nanomembrane. The inset below depicts rays which propagate within the waveguide at various angle. (b) The loss of each discretized angle and cumulative loss. A larger incident angle results in great increased losses due to absorption. The cumulative loss is calculated by adding up the power contributions of each angle. (c) The number of rays in relation to the number of reflections on Mo. The inset illustrates how the number of reflections varies for rays starting at different incident angels.

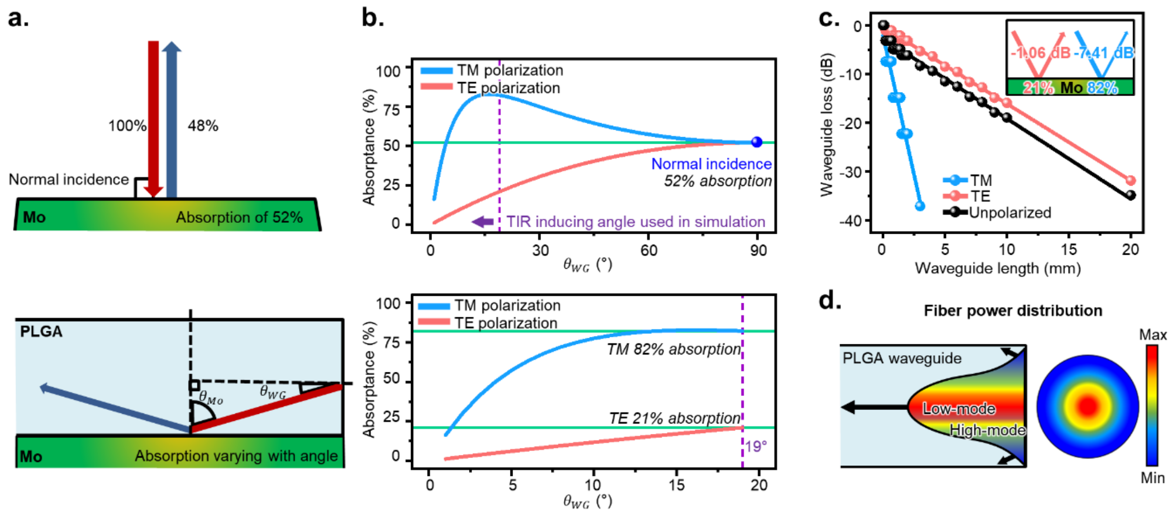

Supplementary Figure S8 | The changing absorption of Mo with the incident angle. (a) Schematic of normal incidence (top) and oblique incidence in the proposed waveguide (bottom). The absorption rate changes with the θ_{Mo}, that is determined by the θ_{WG} at entrance of the waveguide. (b) Absorption spectra for θ_{WG}. In all possible range of incident angle (top), normal incidence to the Mo layer is indicated by the blue dot. The range used in the simulation, which is left side by the violet dashes, is plotted separately (bottom). (c) Losses of waveguide for two polarization states and unpolarized state. Simulation was conducted in a single-channel waveguide with θ_{WG} of 19°. The inset image shows absorption loss for a single reflection in both polarizations, respectively. (d) Power distribution of optical fibers. The low-order modes at the center occupy substantial amount of power and deliver considerable power.

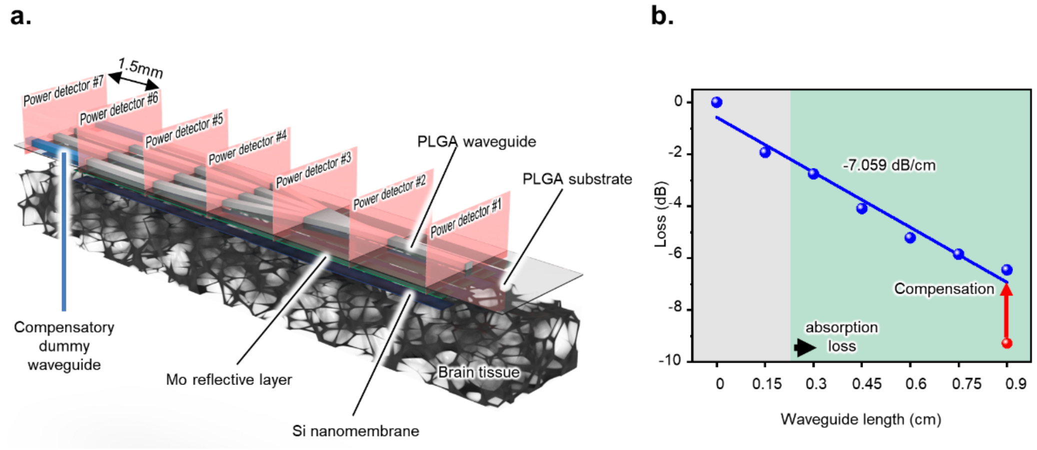

Supplementary Figure S9 | The actual 4-channel waveguide simulation with Mo layer. (a) Simulation system and detector arrangement for loss measurement. The blue segment at the end of waveguide is an extension of short waveguide to compensate the length mismatch. (b) Loss of the compensated waveguide. Absorption losses occur after where the waveguide overlaps with the Mo layer, indicated green area.

a.

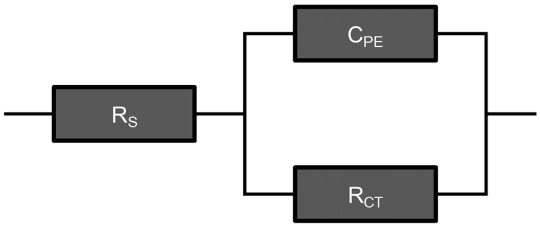

	Mo/Si	Au
R_{CT} (M-ohms)	902.9	38.1
C_{PE} ($uFcm^{-2}$)	1.98	3.65

b.

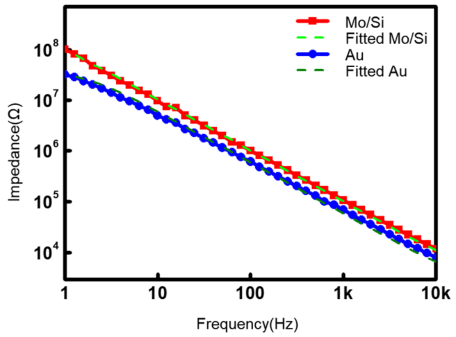

Supplementary Figure S10 | EIS characterizations of Mo/Si and Au electrodes. (a) Diagram of the equivalent Randles circuit model applied to fit the EIS measurements and representative values of the C_{PE} and R_{CT} for Mo/Si and Au electrodes, where C_{PE} is the double-layer capacitance, R_{CT} is the charge transfer resistance, and R_S is the solution resistance. (b) Impedance measurement results (solid lines) and fitted data (dashed lines).

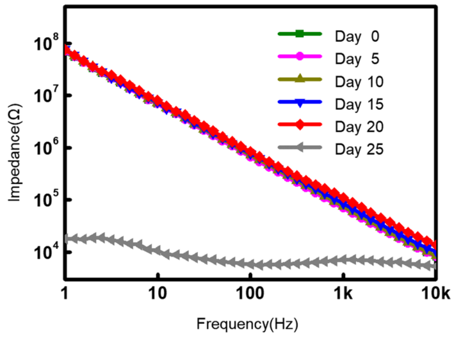

Supplementary Figure S11 | Prolonged stability test of SiO_2 encapsulation layer. Electrochemical impedance changes of a Mo/Si electrode with a 150nm-thick SiO_2 encapsulation layer were observed in a phosphate-buffered saline solution at pH 7.4 at 37 °C over a period of 25 days.

Supplementary Note 1. Details in equations for TIR conditions

(1) Calculation of critical angle for TIR

$$\theta_{TIR,c} = \sin^{-1}\left(\frac{n_{biofluid}}{n_{PLGA}}\right) = 69.8°$$

Thus, TIR can occur, when $\theta_{TIR} \geq 69.8°$

(2) Calculation of critical light emitting angle for TIR

$$n_{OA}\, sin(\theta_{in}) = n_{PLGA}\, sin\left(\frac{\pi}{2} - \theta_{TIR}\right)$$

$$sin(\theta_{in}) = \frac{n_{PLGA}}{n_{OA}} cos(\theta_{TIR})$$

$$\theta_{in} = sin^{-1}\left(\frac{n_{PLGA}}{n_{OA}} cos(\theta_{TIR})\right)$$

So, $\theta_{in,c}$ = 19.5° and θ_{in} should be smaller than 19.5° for TIR, when n_{OA} = 1.51.

(3) Calculation of reflectance at oblique angle

$$r_{S-pol} = \frac{n_{PLGA}\cos\theta - n_{Biofluid}\left[1-\left(\frac{n_1}{n_2}\right)^2 \sin^2(\theta)\right]^{\frac{1}{2}}}{n_{PLGA}\cos\theta + n_{Biofluid}\left[1-\left(\frac{n_1}{n_2}\right)^2 \sin^2(\theta)\right]^{\frac{1}{2}}} \text{ and } R_{S-pol} = r_{S-pol} \cdot r_{S-pol}^*$$

$$r_{P-pol} = \frac{n_{Biofluid}\cos\theta - n_{PLGA}\left[1-\left(\frac{n_1}{n_2}\right)^2 \sin^2(\theta)\right]^{\frac{1}{2}}}{n_{Biofluid}\cos\theta + n_{PLGA}\left[1-\left(\frac{n_1}{n_2}\right)^2 \sin^2(\theta)\right]^{\frac{1}{2}}} \text{ and } R_{P-pol} = r_{P-pol} \cdot r_{P-pol}^*$$

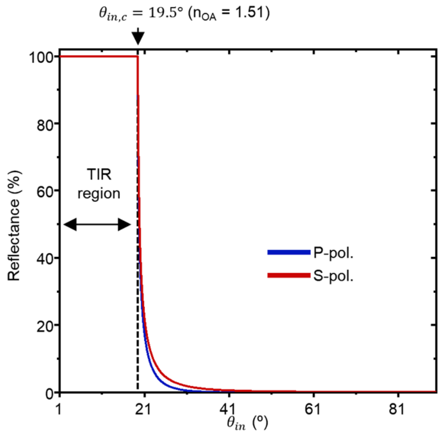

Supplementary Figure S12 | Reflectance curve for S- and P-polarized light as a function of $\boldsymbol{\theta_{in}}$. The θ_{in} should be below 19.5 degree to maintain TIR in PLGA waveguide.

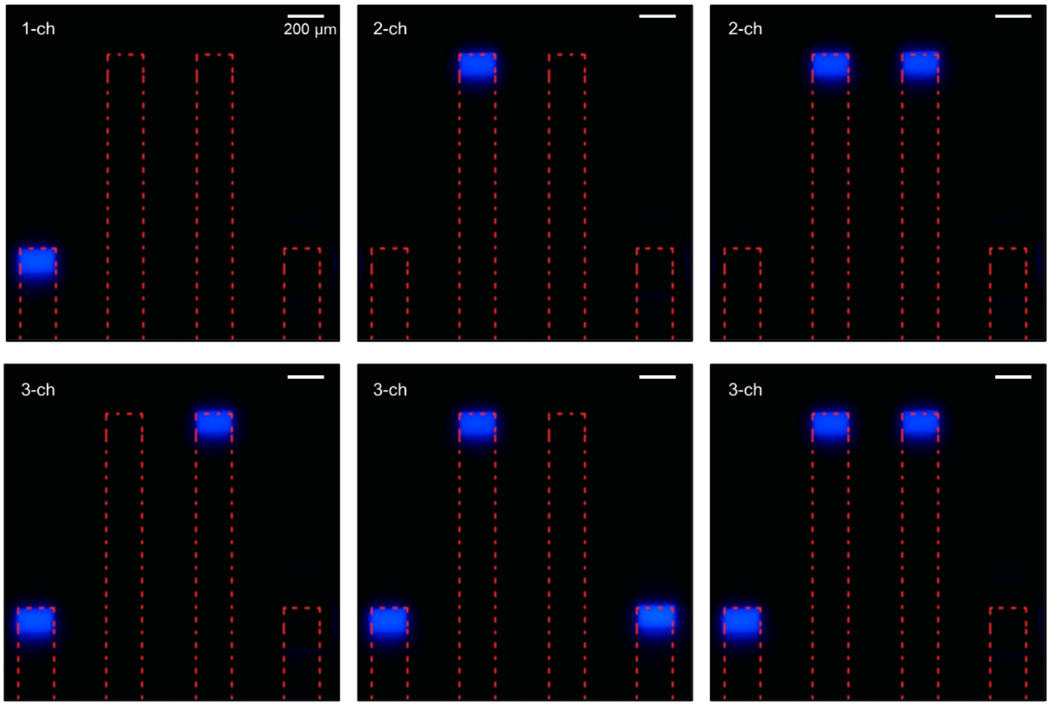

Supplementary Figure S13 | PLGA waveguides with various form factors fabricated through soft lithography showing examples of individually fabricated 1-ch, 2-ch, and 3-ch waveguides.

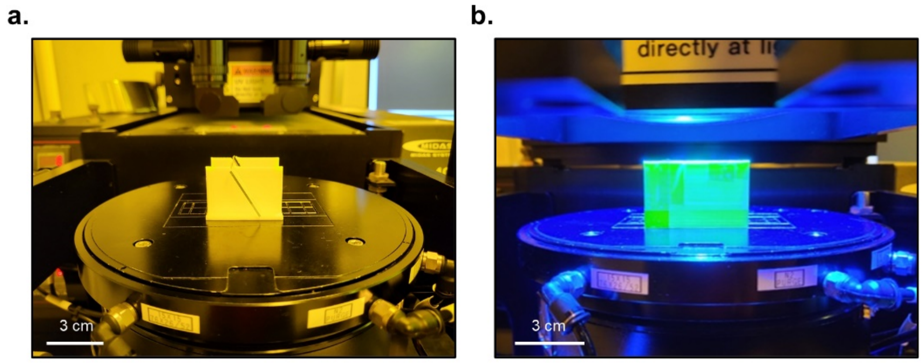

Supplementary Figure S14 | Photographs of the jig for making the master mold. (a) Master mold with 60° inclined slits. (b) Photograph of UV irradiation on the master mold fixed to the jig.

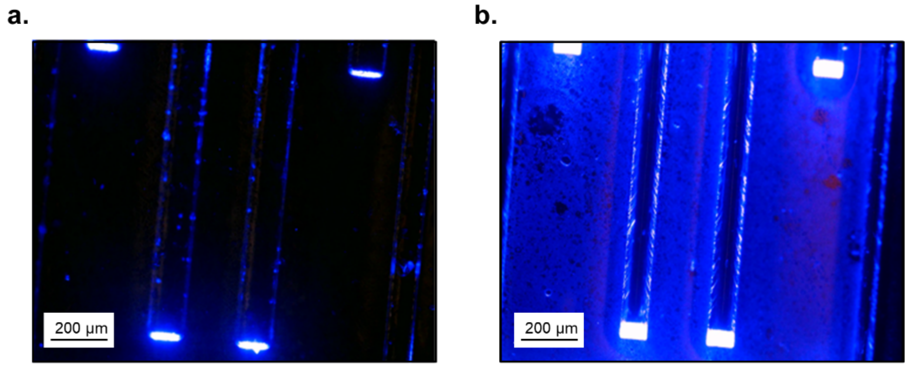

Supplementary Figure S15 | Optical microscope images of the 4-ch PLGA waveguide. Emissions of 460 nm blue light from (a) 90° and (b) 50° angled tips.

a.

Simulated gray level images after noise filtering

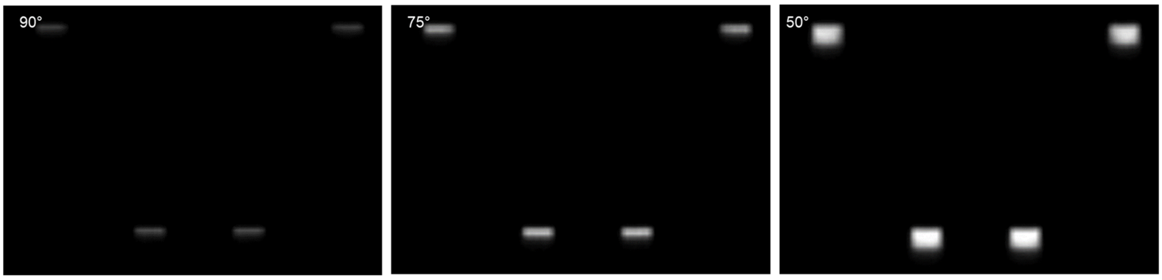

b.

Experimental gray level images after noise filtering

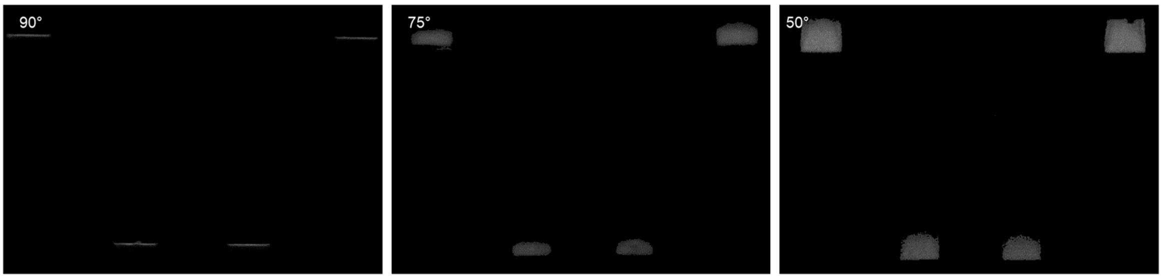

Supplementary Figure S16 | Grayscale images of the PLGA waveguide tips emitting blue light. Results of grayscale-converted and noise-filtered images applied to (a) simulated and (b) experimental data based on the angles of the waveguide tips.

a.

Simulated results

**Angle**	90	75	50
**Mean intensity**	26.6440	55.2658	89.3150
**Normalization**	1	2.0742	3.3522

b.

Experimental results

**Angle**	90	75	50
**Mean intensity (n=4)**	17.0499	33.1965	46.1859
**Normalization**	1	1.9470	2.7089

Supplementary Figure S17 | Quantitative comparisons of the gray level images. Results of quantitative comparisons of the (a) simulated and (b) measured results based on the angles of the waveguide tips.

Fill Factor(%)	63%	56%	42%
R_{CT} (M-ohms)	568.9	659.2	856.9
C_{PE} ($uFcm^{-2}$)	3.14	2.75	1.95

Supplementary Figure S18 | Representative C_{PE} and R_{CT} values for the grid-shaped Mo/Si electrodes fitted to the equivalent Randles circuit model for different fill factors.

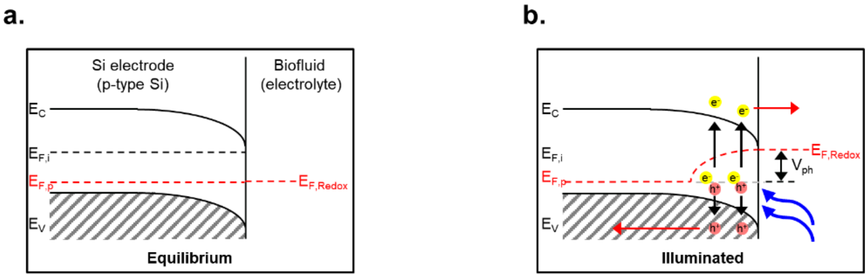

Supplementary Figure S19 | Energy band diagrams at the p-Si electrode and biofluid interface under (a) equilibrium and (b) illuminated conditions.

Power (mW) [Optical fiber]	Intensity ($mWmm^{-2}$) [Optical fiber]
1	126.12
2	252.25
5	630.62
10	1261.24
20	2522.48
30	3782.72

Supplementary Figure S20 | Power and intensity values of the 460 nm blue light emitted from the optical fiber as well as the practical light intensity for stimulating the cortical surface via optical fiber emission.

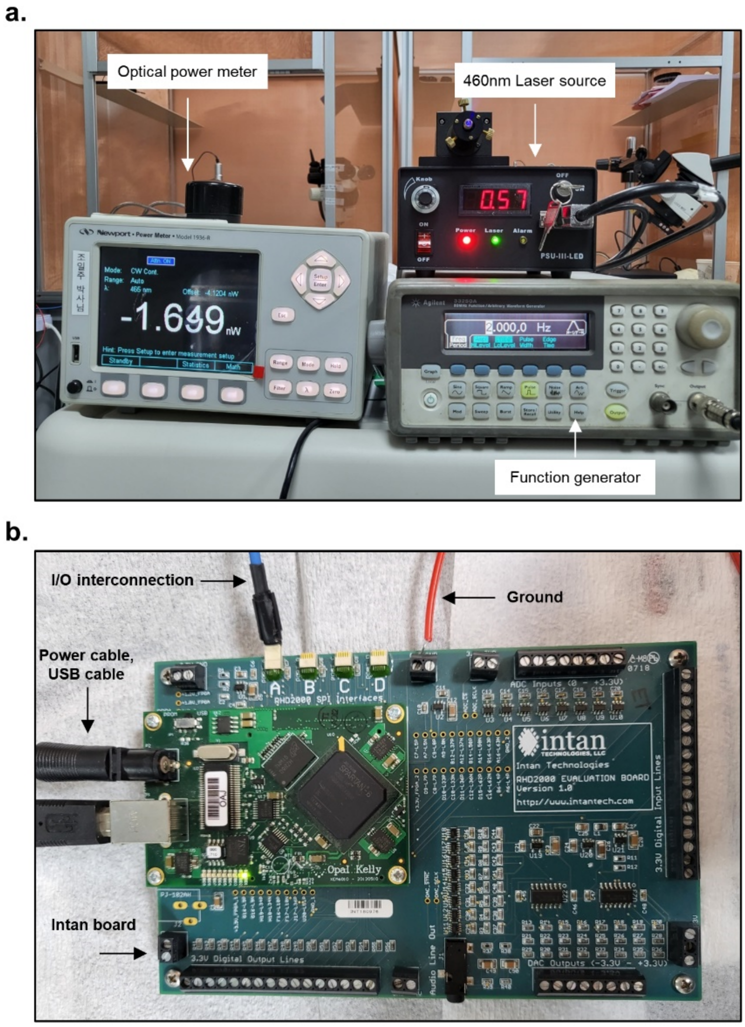

Supplementary Figure S21 | Detailed experimental equipment images for in vitro and in vivo experiments. (a) Photograph of the optical power meter for light power setting, with 460 nm laser source and function generator for pulse generation. (b) Photograph of Intan equipment for photoinduced artifact and ECoG recording.

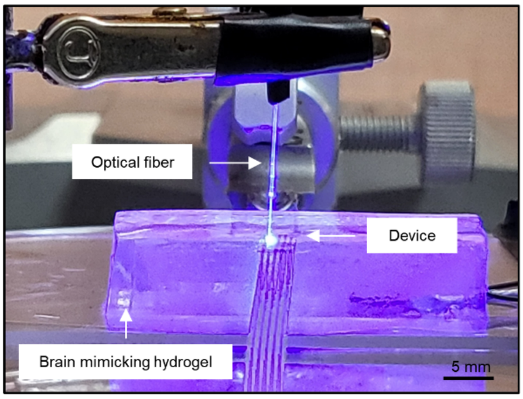

Supplementary Figure S22 | Experimental setup for in vitro photoinduced artifact evaluation.

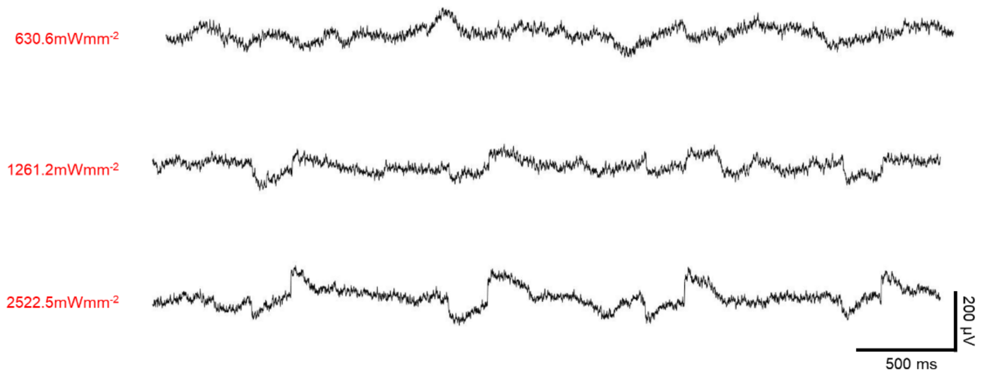

Supplementary Figure S23 | In vitro recorded artifact signals from Mo/Si electrodes according to illuminating light intensity.

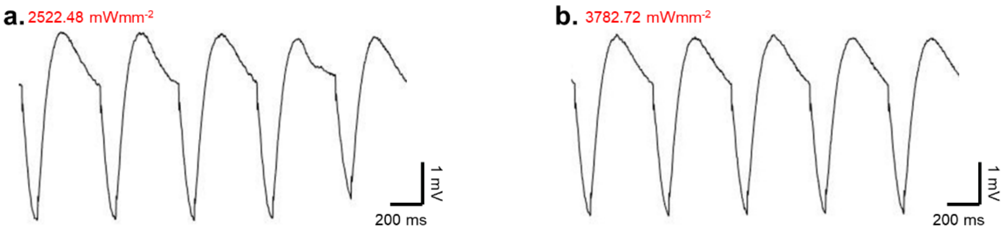

Supplementary Figure S24 | Photoinduced artifact on monolayer Si nanomembrane electrodes. The Si electrode was subjected to laser pulses with intensities of (a) 2522.48 $mWmm^{-2}$ and 3782.72 $mWmm^{-2}$.

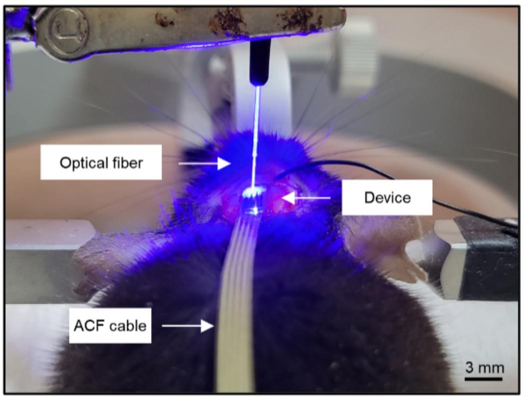

Supplementary Figure S25 | Experimental setup for in vivo photoinduced artifact evaluation.

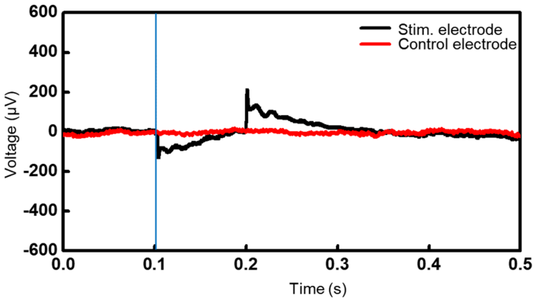

Supplementary Figure S26 | In vivo recorded artifact signals from Mo/Si electrodes with blue light illumination of intensity at 2522.48 mWmm^{-2}.

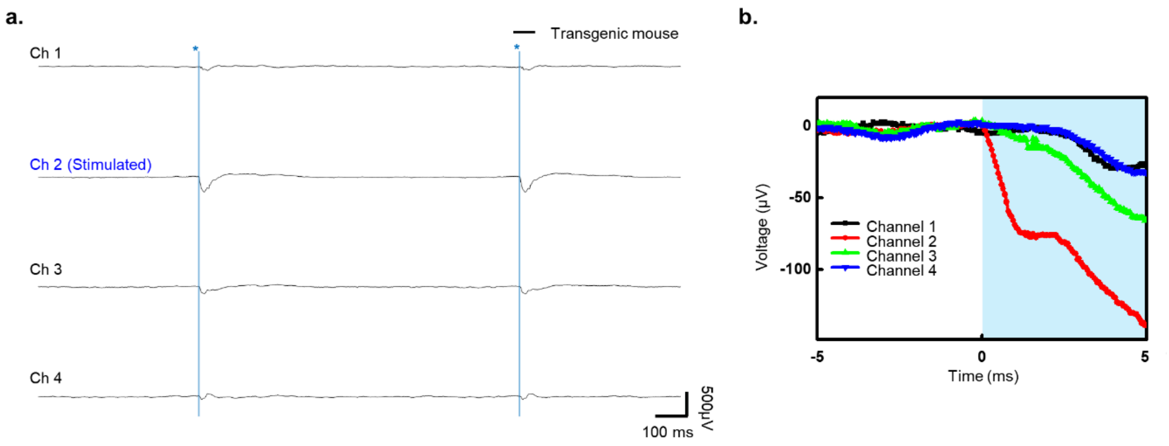

Supplementary Figure S27 | ECoG including evoked LFPs recorded by 4-channel electrode array from single channel pulsed photo-stimulation via optical fiber on transgenic mice. a, Recorded LFP signal from Thy-1: ChR2 mouse. Stimulation with 460 nm pulses (intensity: 63.08 $mWmm^{-2}$, duration: 30 ms, frequency: 1 Hz) is applied exclusively at the electrode of channel 2. The blue line indicates the instant of stimulation. **b,** Sorted optogenetic LFP responses recorded from the 4-ch electrode array. The stimulation occurs at 0 ms.

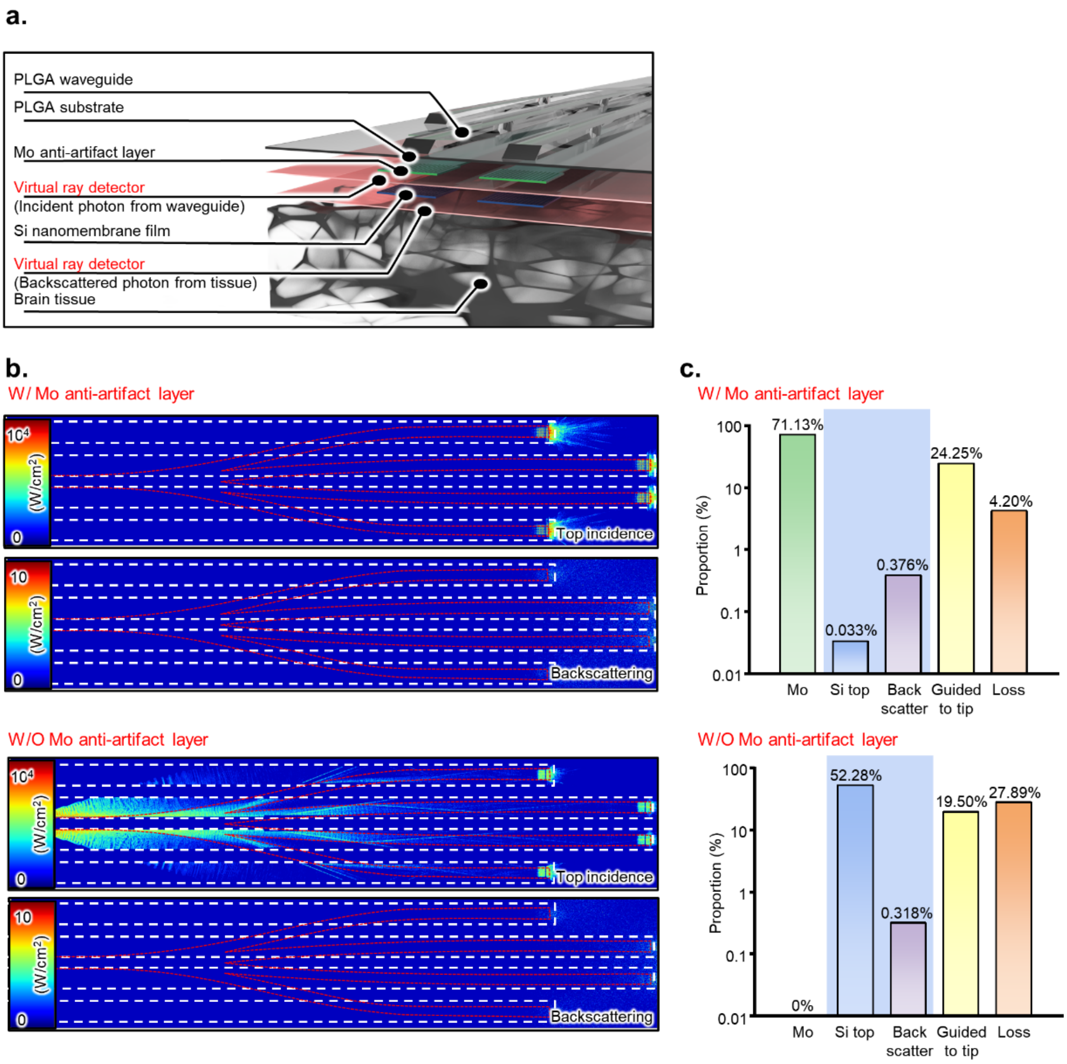

Supplementary Figure S28 | Results depicting photon absorption with and without Mo layer. (a) Schematic representation of the simulation setup with two ray detectors positioned at the upper and lower surfaces of the Si film to measure the amount of photon absorption from both directions. (b) Contour plots for absorption profiles with/without Mo anti-artifact layer captured by ray detectors, indicating photon incidence from waveguide and backscattering from tissue. Si nanomembranes are highlighted by white dashed lines and PLGA waveguide is denoted by red dashed lines. (c) Proportion of photons absorbed or transmitted within the system with/without Mo anti-artifact layer. The blue region indicating the total absorption of Si nanomembrane.

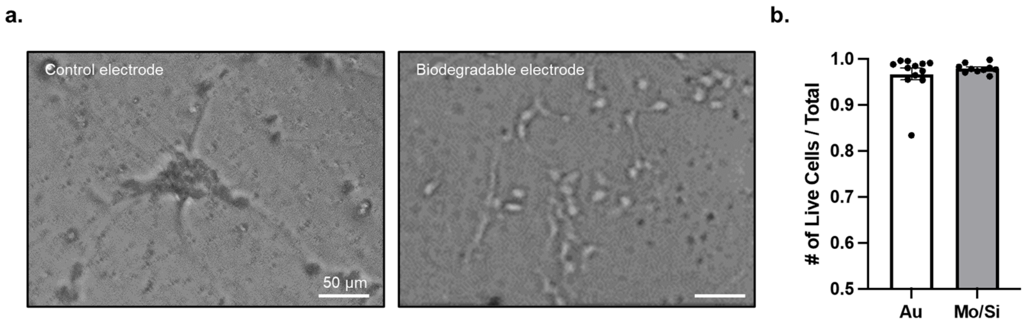

Supplementary Figure S29 | Results of cell viability test. (a) Representative images indicating healthy condition of the cultured primary hippocampal neuronal cells on both the control and biodegradable electrode arrays. (b) Quantitative analyses of the live/dead assays on the control (blue) and bioresorbable (yellow) samples using Invitrogen™ EVOS™ digital color fluorescence microscope (Au: mean, s.e.m., Mo/Si: mean, s.e.m.) (n = 12, 10, where n is each sample number).

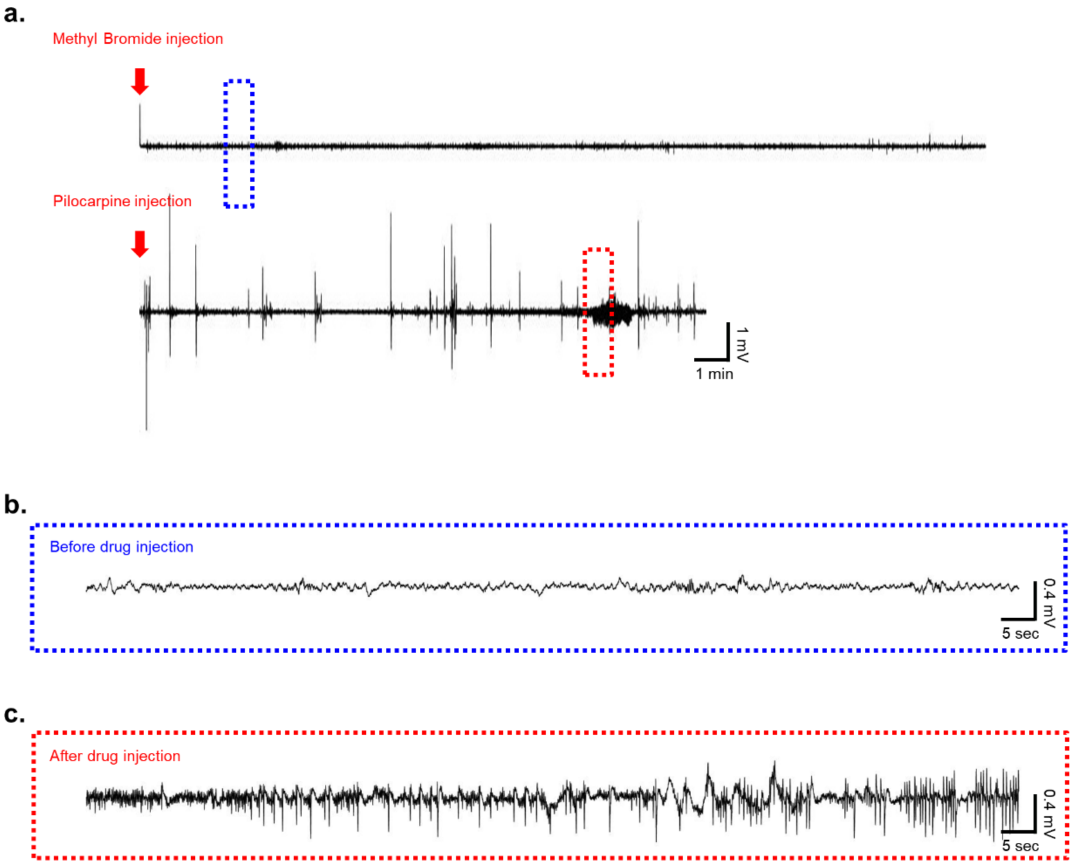

Supplementary Figure S30 | Results of the in vivo ECoG recording experiments including the hybrid bioresorbable system. (a) Recorded ECoG from the mouse over time during (b) methyl bromide and (c) pilocarpine administration.

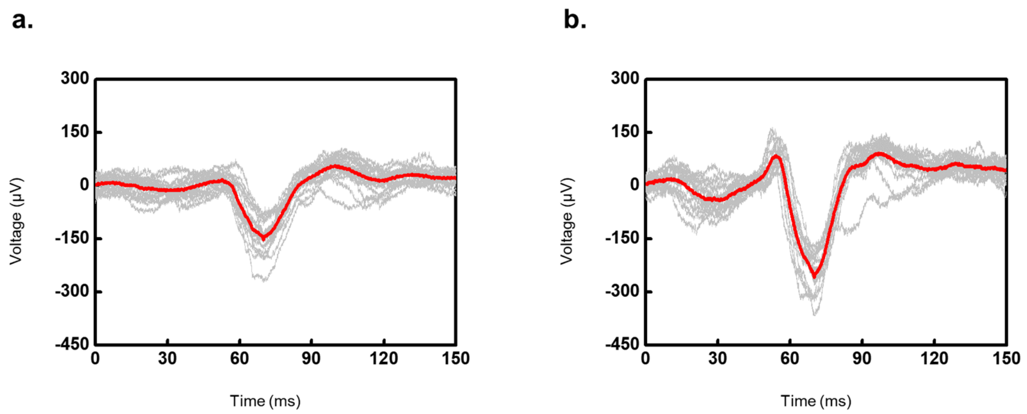

Supplementary Figure S31 | single-spike sorting of recorded seizure-like spiking activity. Spikes (a) recorded from Mo/Si electrode and (b) recorded from Au electrode.

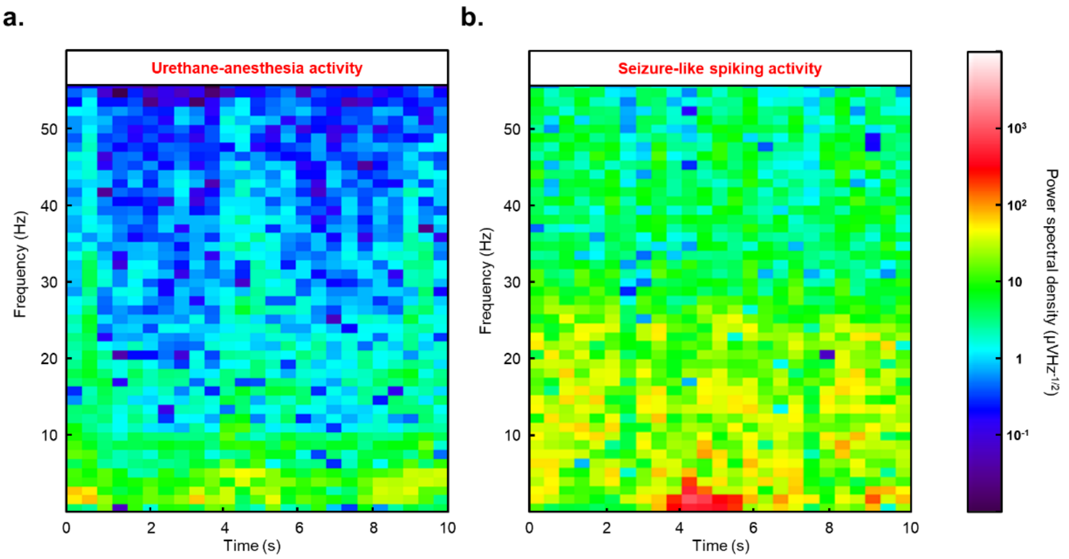

Supplementary Figure S32 | Spectrogram of evoked LFP power spectral density in the frequency range of 0–60 Hz. Spectrograms of (a) urethane-anesthesia and (b) seizure-like spiking activities.

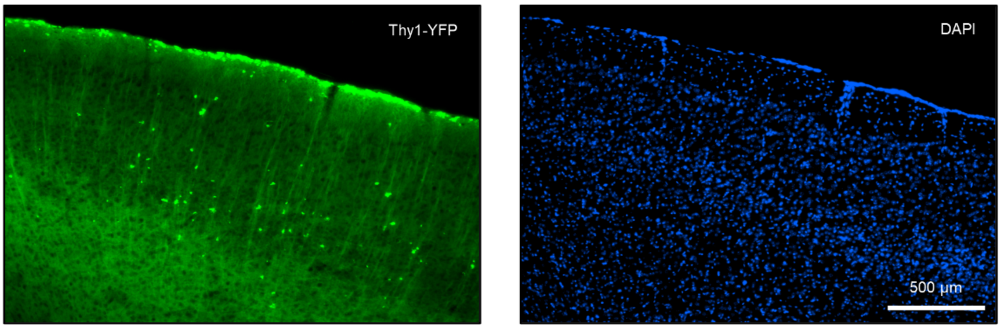

Supplementary Figure S33 | Fluorescence images of cortical area from Thy-1: ChR2 transgenic mice (YFP). Cell nuclei are visualized with DAPI stain (blue). (scale bars = 500 µm)

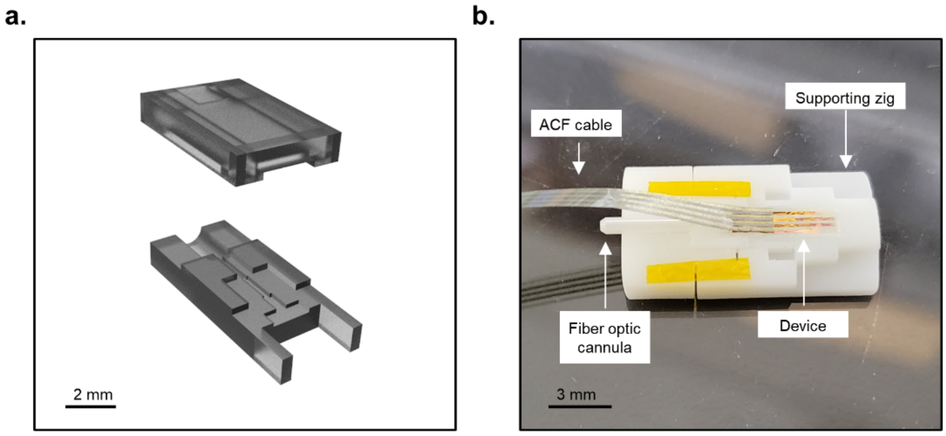

Supplementary Figure S34 | Head-stage design for device implantation. (a) 3D rendering model of head-stage. (b) A photograph of the head-stage and device integration with supporting zig.

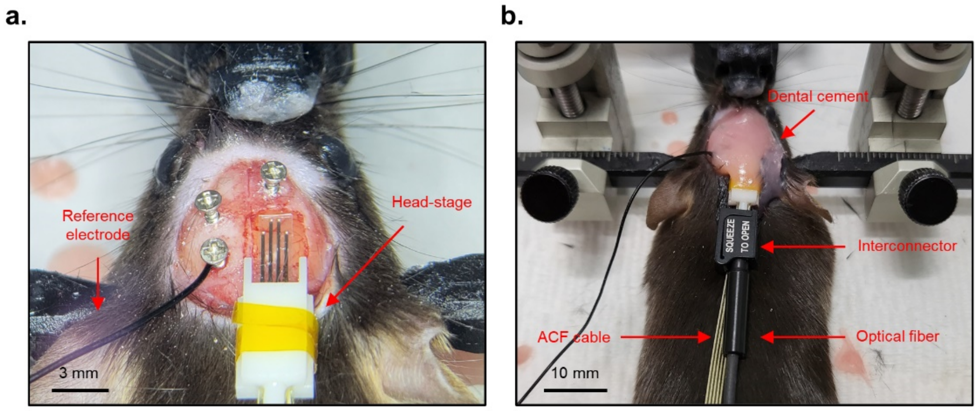

Supplementary Figure S35 | Experimental setup for in vivo ECoG recording and optogenetic stimulation. (a) Photograph of the cranial window for in vivo recording and stimulation experiments before applying the dental cement. (b) Photograph after fully implanting the device.

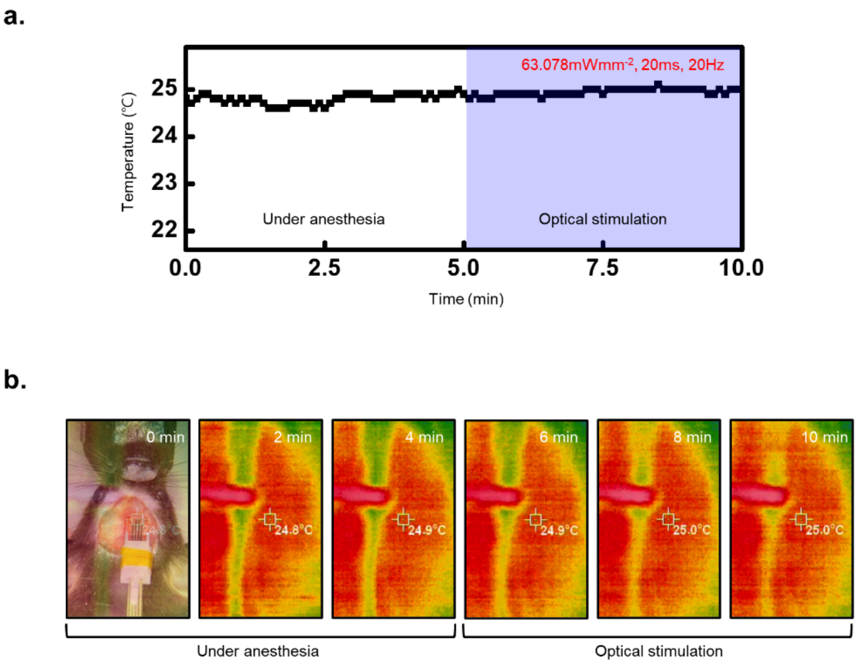

Supplementary Figure S36 | Monitoring temperature of the cerebral cortex and the implanted device. (a) Polt of the temperature in the stimulation area before and during a 5-minute optical stimulation. The stimulation condition is a 460nm blue light pulse with an intensity of 63.08 mWmm^{-2}, a duration of 20 ms, and a frequency of 20 Hz. (b) Infrared thermal images collected at 2-minute intervals. The white crosshair aims the stimulation area.

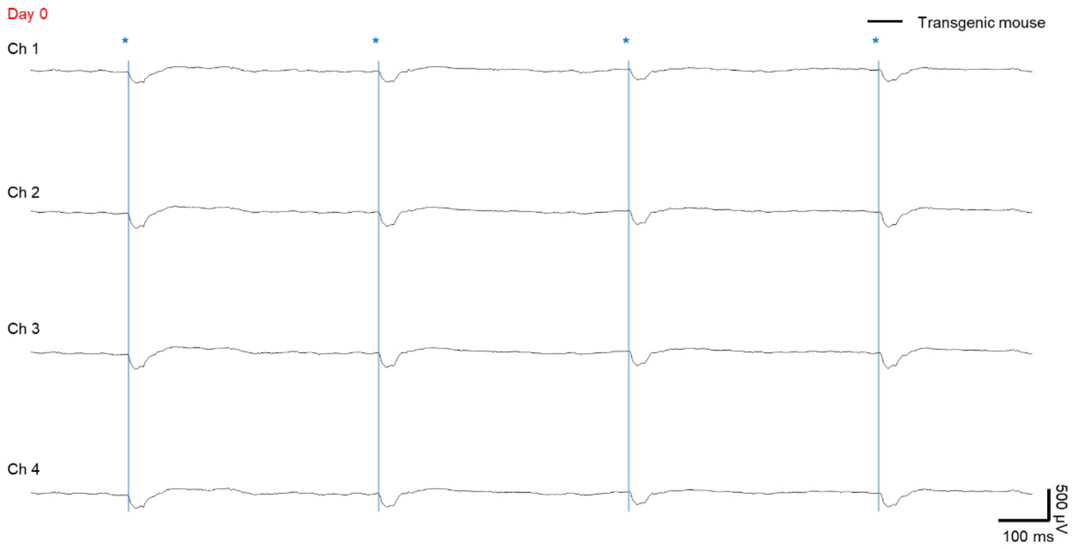

Supplementary Figure S37 | Recorded ECoG from the cerebral cortex of the transgenic mouse upon stimulation with 460 nm blue light pulses(intensity: 63.08mWmm^{-2}, frequency: 2 Hz, duration: 30ms).

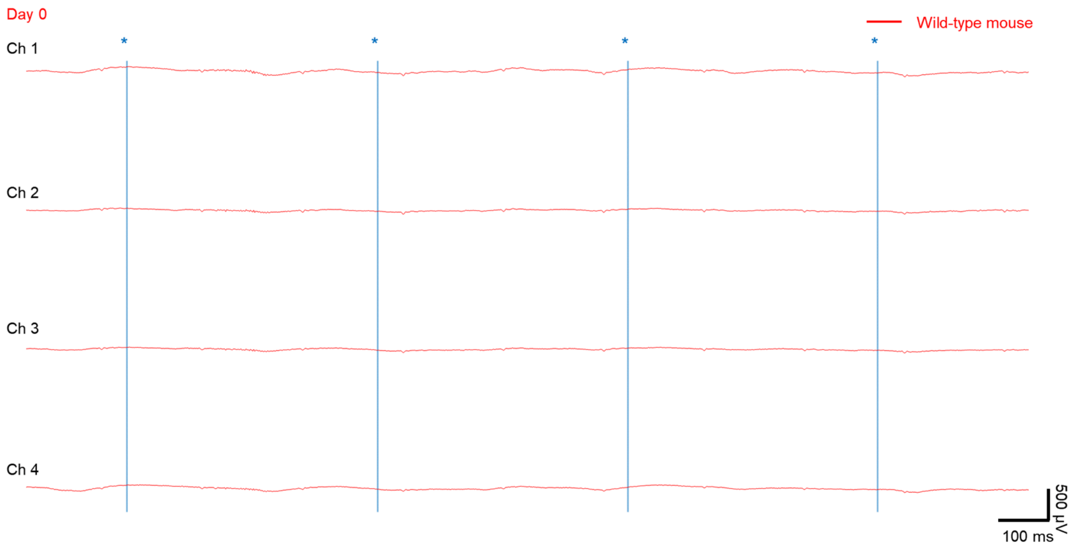

Supplementary Figure S38 | Recorded ECoG from the cerebral cortex of the wild-type mouse upon stimulation with 460 nm blue light pulses(intensity: 63.08mWmm^{-2}, frequency: 2 Hz, duration: 30ms).

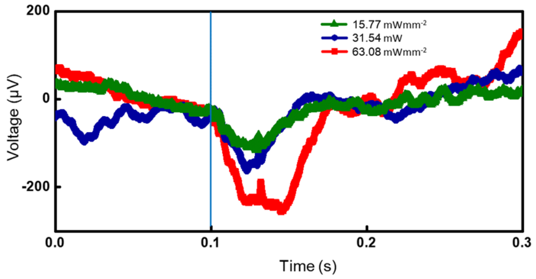

Supplementary Figure S39 | Recorded LFPs from the cerebral cortex of the Thy-ChR2-YFP mouse upon stimulation with 460 nm blue light pulses (duration: 30 ms, frequency: 2 Hz) based on the stimulation intensity.

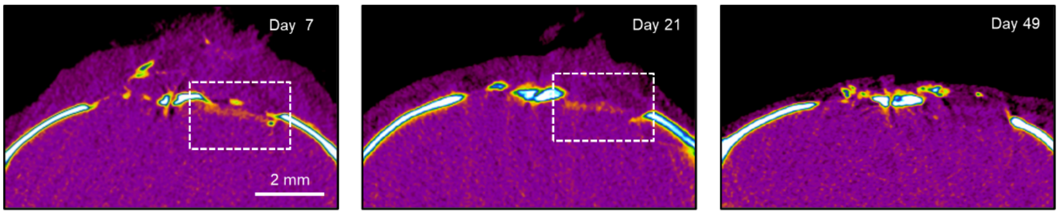

Supplementary Figure S40 | Dissolution characteristic of the implanted device. Computed tomography images of coronal section of the mouse skull collected over 49 days following the device implantation. The white dotted box highlights the bioresorbable device.
