# Supplementary material for: Fully bioresorbable hybrid opto-electronic neural implant system for simultaneous electrophysiological recording and optogenetic stimulation
Source: Nat Commun. 2024 Mar 6;15:2000. doi: 10.1038/s41467-024-45803-0 (PMC10917781; doi:10.1038/s41467-024-45803-0)
Supplement: Supplementary file 3 — Reporting Summary [file 41467_2024_45803_MOESM3_ESM.pdf]

nature portfolio

Corresponding author(s): Ki Jun Yu, Il-Joo Cho, Gil Ju Lee

Last updated by author(s): Jan 30, 2024

Reporting Summary

Nature Portfolio wishes to improve the reproducibility of the work that we publish. This form provides structure for consistency and transparency in reporting. For further information on Nature Portfolio policies, see our Editorial Policies and the Editorial Policy Checklist.

Statistics

For all statistical analyses, confirm that the following items are present in the figure legend, table legend, main text, or Methods section.

n/a	Confirmed	
☐	☒	The exact sample size (*n*) for each experimental group/condition, given as a discrete number and unit of measurement
☐	☒	A statement on whether measurements were taken from distinct samples or whether the same sample was measured repeatedly
☐	☒	The statistical test(s) used AND whether they are one- or two-sided<br>*Only common tests should be described solely by name; describe more complex techniques in the Methods section.*
☐	☒	A description of all covariates tested
☒	☐	A description of any assumptions or corrections, such as tests of normality and adjustment for multiple comparisons
☐	☒	A full description of the statistical parameters including central tendency (e.g. means) or other basic estimates (e.g. regression coefficient) AND variation (e.g. standard deviation) or associated estimates of uncertainty (e.g. confidence intervals)
☐	☒	For null hypothesis testing, the test statistic (e.g. *F*, *t*, *r*) with confidence intervals, effect sizes, degrees of freedom and *P* value noted<br>*Give P values as exact values whenever suitable.*
☒	☐	For Bayesian analysis, information on the choice of priors and Markov chain Monte Carlo settings
☒	☐	For hierarchical and complex designs, identification of the appropriate level for tests and full reporting of outcomes
☒	☐	Estimates of effect sizes (e.g. Cohen's *d*, Pearson's *r*), indicating how they were calculated

Our web collection on statistics for biologists contains articles on many of the points above.

Software and code

Policy information about availability of computer code

Data collection	Electrochemical impedance spectroscopy measurements were obtained using a built-in software of Gamry Reference 600+ potentiostat (Gamry Instruments). Transmittance were measured using a built-in software of UV/VIS spectrophotometer (V-650, JASCO). all of in vitro and in vivo electrophysiological signals were measured using a built-in software of Intan system (RHD 2000 EVALUATION BOARD Version 1.0, Intan Technologies) and collecting MATLAB RHD file reader software(Intan Technologies).
Data analysis	All plot including the electrochemical impedance plots and the transmittance plots were depicted as the mean by using Origin Pro 8.10 (Origin Lab) software. OpticStudio 16.5 was used as a commercial software for simulating all ray-tracing simulations. We developed custom MATLAB scripts using the built-in library for processing calculating incident angle range for waveguiding. Intan RHX was used as a commercial software to analyze the power spectral density of measured electrocorticograms.

For manuscripts utilizing custom algorithms or software that are central to the research but not yet described in published literature, software must be made available to editors and reviewers. We strongly encourage code deposition in a community repository (e.g. GitHub). See the Nature Portfolio guidelines for submitting code & software for further information.

Data

Policy information about availability of data

All manuscripts must include a data availability statement. This statement should provide the following information, where applicable:

- Accession codes, unique identifiers, or web links for publicly available datasets
- A description of any restrictions on data availability
- For clinical datasets or third party data, please ensure that the statement adheres to our policy

Experimental data were generated by our team using microscope measurement. Our research involved raw image data in the form of image files, which converted into text files. Our raw data are publicly available on the Zenodo repository (https://zenodo.org/record/xxxxxx). The raw image data were preprocessed using custom MATLAB scripts to remove noise and normalize the intensities.

Research involving human participants, their data, or biological material

Policy information about studies with human participants or human data. See also policy information about sex, gender (identity/presentation), and sexual orientation and race, ethnicity and racism.

Reporting on sex and gender	N/A
Reporting on race, ethnicity, or other socially relevant groupings	N/A
Population characteristics	N/A
Recruitment	N/A
Ethics oversight	N/A

Note that full information on the approval of the study protocol must also be provided in the manuscript.

Field-specific reporting

Please select the one below that is the best fit for your research. If you are not sure, read the appropriate sections before making your selection.

- [x] Life sciences
- [ ] Behavioural & social sciences
- [ ] Ecological, evolutionary & environmental sciences

For a reference copy of the document with all sections, see nature.com/documents/nr-reporting-summary-flat.pdf

Life sciences study design

All studies must disclose on these points even when the disclosure is negative.

Sample size	For cell viability experiments involving quantification of puncta, puncta intensity, and numbers of signal positive cells (green) in cultured neurons, n=10 was chosen as the minimal replicate number, and sample size was determined by the number positive cells (green) within the replicates. We determined this to be sufficient owing to specific staining of positionally defined cell types using known markers.
Data exclusions	Data were not excluded from analysis.
Replication	All replication attempts were successful. For quantification, all samples were quantified for a minimum of three electrodes, where quantification of cell puncta (green) are specific to the image shown.
Randomization	Electrodes used for imaging were selected randomly, however all cells passed quality control and analyzed equally. All images were repeatedly selected blind to the experimental group for fluorescence intensity calculation.
Blinding	Blinding was not possible as experimental conditions were evident from the image data.

Reporting for specific materials, systems and methods

We require information from authors about some types of materials, experimental systems and methods used in many studies. Here, indicate whether each material, system or method listed is relevant to your study. If you are not sure if a list item applies to your research, read the appropriate section before selecting a response.

Materials & experimental systems

n/a	Involved in the study
☐	☒ Antibodies
☒	☐ Eukaryotic cell lines
☒	☐ Palaeontology and archaeology
☐	☒ Animals and other organisms
☒	☐ Clinical data
☒	☐ Dual use research of concern
☒	☐ Plants

Methods

n/a	Involved in the study
☒	☐ ChIP-seq
☒	☐ Flow cytometry
☒	☐ MRI-based neuroimaging

Antibodies

Antibodies used: Chicken-anti-GFAP, 1:500, AB5541, EMD Miliipore Corp.; Rabbit-anti-Iba1, 1:200, 019-19741, Wako; donkey-anti-chicken conjugated Alexa Fluor 488, 1:500, #703-545-155, Jackson; donkey-anti-rabbit conjugated Alexa Fluor 594, 1:500, #711-585-152, Jackson

Validation: We purchased antibodies from Millipore Cop., Wako, and Jackson.

Animals and other research organisms

Policy information about studies involving animals; ARRIVE guidelines recommended for reporting animal research, and Sex and Gender in Research

Laboratory animals: The experiments utilized adult male wild mice (C57BL/6), Thy-1: ChR2 transgenic mice (C57BL/6) (age: 8-10 weeks), and Sprague–Dawley rat embryos (E18).

Wild animals: No wild animals were used in this study.

Reporting on sex: Findings only reported to male animals.

Field-collected samples: No field-collected samples were used in this study.

Ethics oversight: All animal works were performed under the study protocol KIST-IACUC-2022-155, as approved by the Institutional Animal Care and Use Committee of the Korea Institute of Science and Technology (KIST)

Note that full information on the approval of the study protocol must also be provided in the manuscript.

Plants

Seed stocks: N/A

Novel plant genotypes: N/A

Authentication: N/A
